# Insect Antifeedant Components of *Senecio fistulosus* var. *fistulosus*—Hualtata

**DOI:** 10.3390/plants8060176

**Published:** 2019-06-15

**Authors:** Liliana Ruiz-Vásquez, Matías Reina, Víctor Fajardo, Matías López, Azucena González-Coloma

**Affiliations:** 1 Natural Resources Research Center (CIRNA), National University of the Peruvian Amazon (UNAP), Iquitos, Peru; lilyruizv@gmail.com; 2 Institute of Natural Products and Agrobiology (IPNA), Spanish Research Council (CSIC), 38206 Tenerife, Spain; mreina@ipna.csic.es; 3 Faculty of Sciences, University of Magallanes (UMAG), Punta Arenas 01855, Chile; victor.fajardo@umag.cl; 4 University Institute of Bio-Organic Antonio González (IUBO), University of La Laguna, 38206 Tenerife, Spain; mlopez@ull.es; 5 Institute of Agricultural Sciences (ICA), Spanish Research Council (CSIC), 28006 Madrid, Spain

**Keywords:** *Senecio fistulosus*, antifeedant, sesquiterpene, pyrrolizidine alkaloid, structure-activity relationships

## Abstract

From a bioactive methanolic extract of *Senecio fistulosus*, the antifeedant effects of the alkaloidal and non-alkaloidal fractions were tested against the insects *Spodoptera littoralis*, *Myzus persicae* and *Rhopalosiphum padi*, with the non-alkaloidal fraction being antifeedant. The phytochemical study of the non-alkaloidal fraction of *S. fistulosus*, resulted in the isolation of four compounds, two 9-oxo-furanoeremophilanes (**1**, **2**), an eremophilanolide, 1β,10β-epoxy-6-acetoxy-8α-hydroxy-eremofil-7(11)-en-8β,12-olide (**3**) and a maaliol derivative (**4**). The alkaloidal fraction yielded two known pyrrolizidine alkaloids (**5**, **6**). Compounds **1**, **3** and **4** are new natural products. Furanoeremophilane **2** was a strong antifeedant against *S. littoralis* and maaliane **4** inhibited the settling of *M. persicae*.

## 1. Introduction

The genus, *Senecio* (Asteraceae), is distributed worldwide and contains pyrrolizidine alkaloids (PAs). PAs are toxic to mammals and feeding deterrents for insect herbivores [1]. Compounds present in the non-alkaloidal fraction of *Senecio* spp have been described as part of their defense [1,2,3]. The most frequent chemical groups found in the non-alkaloidal fraction of *Senecio* are eremophilane-type sesquiterpenes of the furanoeremophilane and eremophilanolide type [1]. Some of these compounds have insect antifeedant, acaricidal, fungicidal, cytotoxic, phytotoxic, antioxidant, anti-inflammatory, and antimicrobial effects [1,2,3,4,5,6] and have been proposed as being an important part of *Senecio* defense [1,2,3,4,5].

In Chile, the genus *Senecio* is abundant (~210 species) [7]. There are several reports on eremophilane sesquiterpenes from Chilean *Senecio* species with defensive properties [1,2,3]. The species, *Senecio fistulosus*, grows from the western area of Patagonia to central Chile and it is used in folk medicine for its effects on the heart [8,9]. A previous study on the phytochemistry of *S. fistulosus*, from the central region of Chile, reported the presence of furanoeremophilanes 4α-hydroxy-6β-angeloxy-10βacetoxy-9-oxo-furanoeremophilane and 4α-hydroxy-6β-angeloxy-9-oxo-furanoeremophilane [10], but there are no reports on the defensive chemistry of this species.

In this work, the authors studied the chemical defenses of *S. fistulosus* var. *fistulosus* from the Magallanic region, containing a large number of the Chilean *Senecio* species and subspecies distributed in the Patagonic Cordillera and the coastal areas [7].

From a bioactive methanolic extract of *S. fistulosus*, the alkaloidal and non-alkaloidal fractions were tested against the insects *Spodoptera littoralis* (Boisd), *Myzus persicae* (Sulzer) and *Rhopalosiphum padi*, with the non-alkaloidal being antifeedant. Two furanoeremophilanes (**1**, **2**), one eremophilanolide (**3**) and maaliol derivative (**4**) have been isolated from the non-alkaloidal fraction, with compounds **1**, **3** and **4** being reported as natural products for the first time. Additionally, two pyrrolizidine alkaloids (**5**, **6**) were isolated from the alkaloidal fraction.

## 2. Results and Discussion

Extracts of *S. fistulosus* (methanolic, MeOH, non-alkaloidal and alkaloidal) were tested against the phytophagous insects *Spodoptera littoralis, Myzus persicae* and *Rhopalosiphum padi*. The MeOH extract showed a significant effect on *M. persicae* (SI = 78 ± 4%; EC_50_ = 1.69 μg/cm^2^, 0.69–4.15, 95% confidence limits—CL), the non-alkaloidal extract was active on *S. littoralis* (FI = 76 ± 6%) and *M. persicae* (SI = 75 ± 6%; EC_50_ = 2.25 μg/cm^2^, 1.19–4.24, 95% CL). The alkaloidal extract showed moderate activity against *S. littoralis* (SI = 62 ± 6%) and *R. padi* (SI = 69 ± 6%), indicating that *S. fistulosus* defense chemistry is mainly due to compounds present in the non-alkaloidal fraction, as previously suggested for PA producing plants [2,3,4,11].

**Figure 1:**
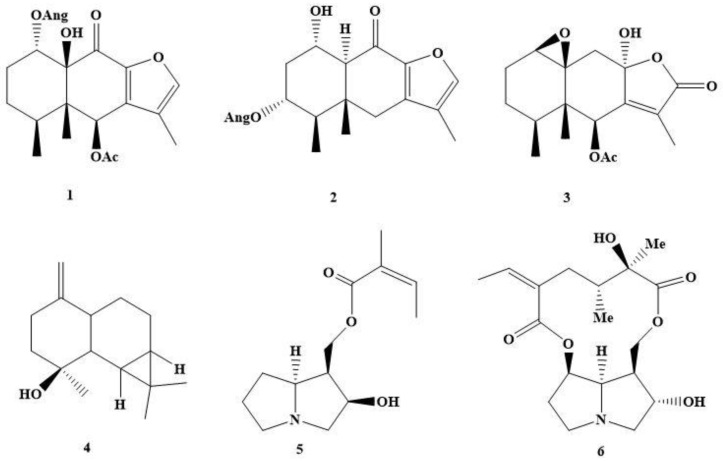
Chemical structures of compounds **1**–**6**.

The phytochemical study of the non-alkaloidal fraction of *S. fistulosus* resulted in the isolation of four compounds, two 9-oxo-furanoeremophilanes (**1**, **2**) [10,12], an eremophilanolide, 1β,10β-epoxy-6-acetoxy-8α-hydroxy-eremofil-7(11)-en-8β,12-olide (**3**) and a maaliol derivative (**4**). The alkaloidal fraction yielded two known pyrrolizidine alkaloids (**5, 6**) [13,14] (Figure 1).

Compounds **1, 3** and **4** are described here for the first time as natural products. A previous study on *S. fistulosus* reported the presence of furanoeremophilanes 4α-hydroxy-6β-angeloxy-10β-acetoxy-9-oxo-furanoeremophilane and 4α-hydroxy-6β-angeloxy-9-oxo-furanoeremophilane [10]. The difference in furanoeremophilane composition could be related to the different origin of the plant populations studied (Magallanes versus the central region of Chile).

The structural elucidation was carried out based on their ^1^H and ^13^C NMR spectra including (1D) and (2D) (COSY, HSQC, HMBC and NOESY) experiments, X-ray diffraction, as well as its physical, spectrometric (EIMS and HREIMS) and comparison with the chemical bibliography reported for similar compounds.

**Figure 2:**
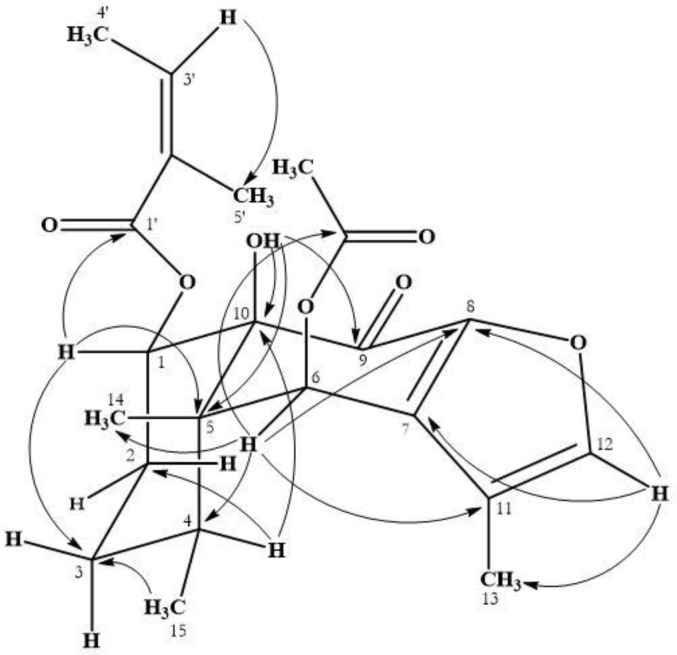
HMBC correlations observed for compound **1**.

**Figure 3:**
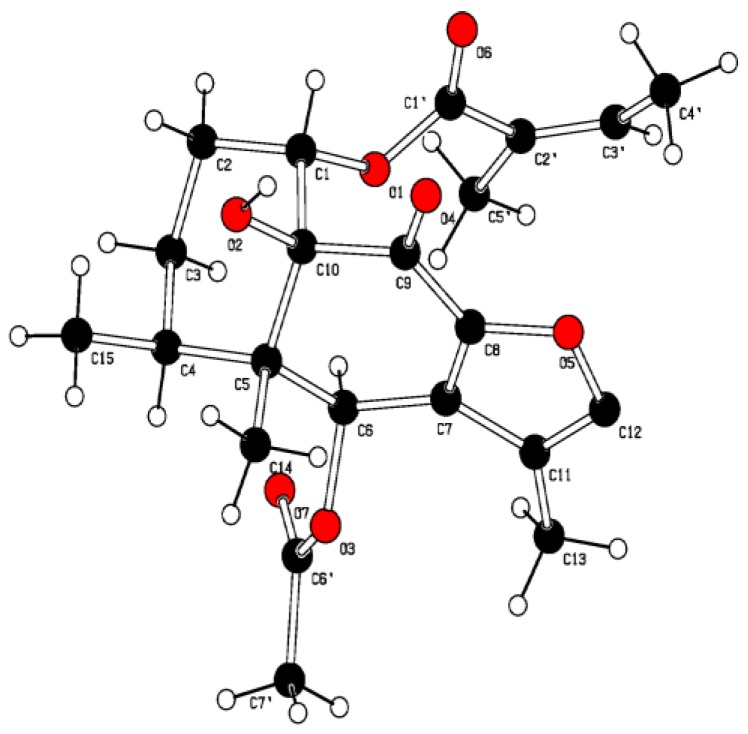
NOESY and ORTEP of compound **1**.

Compound **1** was isolated in crystalline form. Its infrared (IR) spectrum showed absorption bands at 3452, 1748, 1720 and 1679 cm^−1^ attributable to a hydroxyl, ester, and carbonyl groups. Its HR-EI-MS showed a molecular-ion peak at *m/z* 404.1838 (M^+^, calculated for C_22_H_28_O_7_, 404.1835) and a major fragment in the upper part of the spectrum at 345 (3%) [M-OCOCH_3_]^+^. The ^1^H and ^13^C NMR spectra of compound **1** (Table 1) showed signals of an olefinic proton at δ(H) 7.41 (br s, *J* = 1.3, H-C(12)) and one methyl group on a double bond at δ(H) 1.91 (d, *J* = 1.1, Me(13)), indicating the presence of a furan ring with a methyl group at C(11). The chemical shifts of signals δ(H) 0.98 (s), 1.17 (d, *J* = 7.5) assigned to Me(14) and Me(15), indicated a cis-decalin system [10]. Signals at δ(H) 3.98 (br s) were assigned to a proton on a hydroxyl group, and signals at δ(H) 5.92 (qq, *J* = 1.5, 7.2, H-C(3′)), 1.88 (dq, *J* = 1.6, 7.4, Me(4’)), 1.55 (quint., *J* = 1.5 Hz, Me(5’)); δ(C) 126.6 (s, C(2′)), 140.5 (d, C(3′)), 15.8 (q, C(4′)), 19.9 (q, C(5′)) corresponded to an angelate group. The chemical shift at δ(H) 7.03 (s) was attributed to H_α_-(6), a geminal proton of an acetate group [δ(H) 2.19 (s, OCOCH_3_); δ(C) 20.9 (q) and 170.9 (s)]. The HMBC experiment showed correlations between H-C(1) with C(3), C(5), C(10) and C(1′), which allowed for the location of the angelate group at C(1). Correlations of the OH proton with C(5), C(9) and C(10) confirmed the location of the hydroxyl group at C(10). Correlation of H_α_-C(6) with C(4), C(8), C(11), C(14) and OCOCH_3_ located the acetate group at C(6) (Figure 2). The remaining correlations were in agreement with the proposed structure. The relative stereochemistry of **1** was established by a NOESY experiment (Figure 3). H_β_-C(1) gave a positive NOE effect with the H_β_-C(2) and H_β_-C(3) signals, confirming the α-configuration of the angelate group. In the same way, H_α_-C(6) presented a NOE effect with protons H_α_-C(4) and Me(13), establishing the configuration of the acetate group as C-6β. The NOE effect of H-C(3′) with Me(4)′/Me(5′) and the chemical shift of Me(5′) (δ(C) = 20.6 ppm) suggested the Z-geometry for the double bond of the H-C(2´)/H-C(3′) of the angelate group. The molecular structure of **1** was confirmed by X-ray diffraction (Figure 3), resolved by direct methods with SIR97, and was established as 1α-angeloyloxy-6β-acetoxy-10β-hydroxy-9-oxo-furanoeremophilane.

The HR-EI-MS of compound **2**, showed a molecular-ion peak at *m/z* 346.1785 (*M*^+^, calculated for C_20_H_26_O_5_, 346.1780), and its IR spectrum showed the presence of absorption bands at 3446, 1733, 1716 and 1699 cm^−1^ attributable to hydroxyl, ester, and carbonyl groups. The analysis of ^1^H and ^13^C NMR spectroscopic data of **2** (Table 1) indicated the presence of a trans-decalin based on the chemical shift at δ(H) 0.88 (s, Me(14)), and by comparison with published data. Therefore, the structure of **2** was confirmed as 1α-hydroxy-3α-angeloyloxy-10α H-9-oxo-furanoeremophilane, previously isolated from *Senecio smithii*, [12].

**Figure 4:**
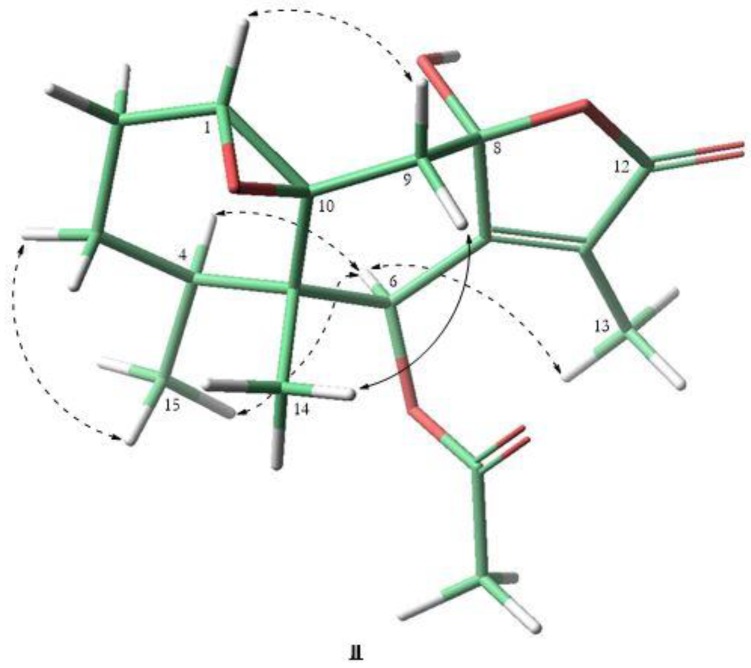
NOESY of compound **3**.

The HR-EI-MS of compound **3** showed a molecular ion peak at *m/z* 322.1425 (M^+^, calculated for C_17_H_22_O_6_, 322.1416) and fragments in the upper part of the spectrum at *m/z* 262 (100%) [M-CH_3_COOH]^+^. Signals for seventeen carbon atoms were observed in its ^13^C NMR spectrum. Their multiplicities were analyzed by a DEPT experiment which determined four methyl, three methylenes, three methines and six quaternary carbons. The ^1^H and ^13^C NMR spectra of **3** showed signals at δ(H) 3.18 (*d*, *J* = 4.6, H-C(1)); δ(C) 62.7 (d, C-(1)) and 60.9 (s, C-(10)), attributed to chemical shifts characteristic of an epoxide group at C-(1)-C-(10); signals at δ(H) 2.20 (s, OCOCH_3_); δ(C) 20.9 (q, OCOCH_3_) and 170.6 (s, OCOCH_3_), corresponding to an acetate group at C(6), a signal for a geminal proton of an acetate group at δ(H) 5.92 (q, *J* = 1.8); δ(C) 73.8 (d, C(6)); and a signal at δ(H) 1.87 (d, *J* = 1.8); δ(C) 8.2 (q) corresponding to a Me(13). The HSQC and HMBC experiments (Table 2) confirmed the presence of an eremophilanolide skeleton and the localization of the epoxide and acetate groups, respectively. The relative stereochemistry of **3** was determined by a NOESY experiment (Figure 4). The positive NOE effect of H_α_-(6) with the methyl Me(13) signal was consistent with a β configuration of the γ-lactone, which agrees with the observed homoalilic coupling constant *J*_6–13_ = 1.8 [15,16] with an angle between the two bonds of about 90°. Therefore, an α-configuration for the hydroxyl group at C(8) was determined. The observed NOE effect of H_α_-(1) with H_β_-(9) confirmed the β-configuration of the epoxide. Compound **3** was identified based on its spectroscopic data as 1β,10β-epoxy-6β-acetoxy-8α-methoxy-eremofil-1(10),7(11)-diene-12,8β-olide, previously obtained by epoxidation and subsequent acetylation of the compound 6β-hydroxy-8α-methoxy-eremophil-1(10),7(11)-dien-12,8β-olide, isolated from *S. magellanicus* [2].

Compound **4** was isolated as a colorless oil. Its HR-EI-MS mass spectrum showed a molecular-ion peak at *m/z* 220.1831 (M^+^, calculated for C_15_H_24_O, 220.1827) and fragments at 205 (55%) [M-CH_3_]^+^ and 187 (19%) [M-CH_3_ + H_2_O]^+^. The presence of three tertiary methyl groups at δ(H) 1.04 (s, Me(13)), 1.06 (s, Me(12)) and 1.28 (s, Me(15)) were observed in the ^1^H and ^13^C NMR spectra (Table 3). An HSQC experiment showed their correlations with carbons at δ(C) 16.5 (q, Me(13)), 28.8 (q, C(12)), and 26.2 (q, C(15)), and signals from two hydrogens of a cyclopropane at δ(H) 0.47 (dd, *J* = 9.6, 11.4, H-C(6)); δ(C) 30.1 (d, C(6)) and 0.71 (ddd, *J* = 6.1, 9.5, 11.4, H-C(7)); δ(C) 27.7 (d, C(7)) [16]. Additional signals attributable to two protons at δ(H) 4.69 (t, *J* = 1.6, H_a_–C(14)) and 4.63 (q, *J* = 1.7 H_b_-C(14)); a δ(C) 106.4 (t) signal assigned to an exocyclic methylene and a proton signal at δ(H) 2.20 (m, H_β_-C(10)); δ(C) 53.6 (d, C(10)) were observed. The latter signal presented HMBC correlations at δ_C_ 153.6 (t, C-(14)), 39.1 (t, C(2)) and 54.6 (d, C(5)), suggesting the location of the exocyclic methylene at C-1. The remaining signals were assigned by analysis of the 1D and 2D NMR spectra and by comparing these data with similar compounds. The structure of **4** was identified as the maaliol derivative (+)-1(14)-en-maaliol [17] which has not been previously isolated as a natural product.

Two unsaturated pyrrolizidine alkaloids (PAs) were isolated from the alkaloidal fraction, 9-O-angeloylpetasinecine (hectorine **5**), and rosmarinine (**6**). These alkaloids were identified by comparison of their spectral data (^1^H a ^13^C NMR and EIMS) with previous reports [13,14].

The antifeedant effects of compounds **1**, **2** and **4** are shown in Table 4. Furanoeremophilane **2** was a strong antifeedant to *S. littoralis* (EC_50_ = 0.64 μg/cm^2^) while the maaliane **4** affected *M. persicae* (EC_50_ = 0.97 μg/cm^2^). Antifeedant furanoeremophilanes have been described in *Senecio* species such as *S. magellanicus* (against *M. persicae* and *S. littoralis*) [2] and *S. otites* (against *M. persicae* and *R. padi*) [11,18].

Furanoeremophilanes are less abundant in *Senecio* than eremophilanolides. Therefore, the studies on their structure-activity relationships (SAR) are limited. Table 5 shows a compilation of the available information on the SAR of these structures, including the results presented in this work. The active compounds against the aphid *M. persicae* are characterized by the absence of substituents in C-1, C-3 and C-10, regardless of the substituent in C-6 (**8–10**, **11**, **12**). The presence of β-OH/C-1 and the α-OAng/C-3 group (compound **2**) resulted in an important antifeedant activity against *S. littoralis*. In addition, the C-6 substitution pattern together with the C-1/C-10 unsaturation determined post-ingestion effects on *S. littoralis* [11].

Maalianes have been isolated from a range of organisms, such as liverworts, marine sponges, soft corals and bacteria, however, they are not abundant in nature. A small amount of biological activity has been reported and includes fish toxicity, in vitro antimalarial activity, cytotoxicity and antimicrobial [19]. This is the first report on the insect antifeedant effects of a maaliane sesquiterpene.

PAs **5** and **6**, with necines of the rosmarinecine and petasinecine type (1,2-saturated base), were isolated from the alkaloidal extract of *S. fistulosus*. The role of PAs as plant defenses against phytophagous insects has been widely documented [20], however, this alkaloidal extract showed moderate-low antifeedant activity (62 ± 6%FR against *S. littoralis* and 69 ± 6%SI against *R. padi*).

PAs with unsaturated retronecines are potentially more toxic than rosmarinecine and petasinecine type (1,2-saturated base) PAs [21]. For example, rosmarinine with a petasinecine, did not form hepatotoxic reactive pyrrole intermediates [22,23] and cytotoxic assays have demonstrated a higher toxicity of retronecine and otonecine PAs compared with platynecine PAs [24]. Therefore, PAs **5** and **6** have a low risk of associated toxicity.

## 3. Materials and Methods

### 3.1. General

For column chromatography (CC), Si-gel (107734, 107741, and 107749, Merck) and Sephadex LH-20 (Sigma–Aldrich) were used. For TLC chromatography, Si-gel (105554 and 105715; Merck) plates were used and visualized with óleum solution (sesquiterpenes) and Dragendorff’s reagent (alkaloids). The prep. HPLC chromatography was carried out on a Beckman 125P system equipped with an Ultrasphere semiprep column (10 × 250 mm) and a UV/visible diode array detector 168. Optical rotations were determined at 20 °C on a Perkin-Elmer 343 Plus polarimeter. IR Spectra were recorded in CHCl_3_ on a Perkin Elmer 1600 spectrophotometer. NMR spectra were recorded on a pulsed-field gradient Bruker Advance II-500 MHz spectrometer (solvent as internal standard CDCl_3_, at δ_H_ 7.26 and δ_C_ 77.0) and the Bruker software was used for DEPT, ^1^H, ^1^H-COSY (Homonuclear correlation spectroscopy), NOESY (Nuclear Overhauser Effect Spectroscopy), HSQC (Heteronuclear single quantum coherence spectroscopy) and HMBC (Heteronuclear Multiple Bond Correlation). EI and HR-EI-MS spectra were recorded in *m/z* on a Micromass Autospec spectrometer.

### 3.2. Extraction and Isolation

Aerial parts of *S. fistulosus* (Asteraceae), identified by Orlando Dollenz, were collected in Sierra Baguales (March 2009, Punta Arenas, Magallanes, Chile,) during the flowering period. A voucher specimen (# 7569) has been deposited in the Herbarium of the Patagonian Institute, Magallanes University (UMAG), Punta Arenas, Chile.

Grounded dried aerial plant parts (2.50 kg) were extracted with MeOH (20 L) at room temperature for a week to give a crude MeOH extract (190.5 g, 7.62% yield of plant dry weight). The MeOH extract (157.6 g) was treated with a f H_2_SO_4_ 0.5 M and CH_2_Cl_2_ (1:1) solution. Zinc dust was used to reduce the aqueous phase under continuous stirring (4–6 hours) and then filtered, basified (30% NH_4_OH, pH = 8–9) and extracted with CH_2_Cl_2_ (236.0 mg of alkaloids, 9.4 × 10^−3^%). The organic phase, dried over anhydrous Na2SO_4_ (non-alkaloidal fraction, 9.0 g, 0.36%), was chromatographed on a SiO_2_ vacuum-liquid chromatography column (VLC) and eluted with a hexane/EtOAc/MeOH gradient to give seven fractions. Fr-0 (hexane 100%, 360.7 mg), Fr-1, (hexane/EtOAc 95:5%, 2.2 g), Fr-2 (hexane/EtOAc 90:10%, 1.7 g), Fr-3 (hexane/EtOAc 75:25%, 1.1 g), Fr-4 (hexane/EtOAc 50:50%, 966.6 mg), Fr-5 (EtOAc 100%, 478.2 mg), Fr-6 (MeOH 100%, 2.6 g). Fr-1 (2.2 g, 8.8 × 10^−2^%) was further chromatographed on a CC Sephadex LH-20 column, CC silica gel and semi-preparative normal-phase HPLC eluted with an isocratic mixture of hexane/EtOAc at 3 ml/min flow rate to give compound **4** (16.1 mg, 6.4 × 10^−4^%). Fr-2 (1.7 g) was chromatographed on CC Sephadex LH-20, CC silica gel, circular chromatography and semi-preparative normal phase HPLC eluted with an isocratic mixture of hexane/EtOAc at a flow rate of 3 ml/min to give compounds **1** (41.0 mg, 1.6 × 10^−3^%), **2** (29.9 mg, 1.2 × 10^−3^%) and **3** (3.5 mg, 1.4 × 10^−4^%).

The alkaloidal fraction was submitted to neutral alumina CC, eluted with an EtOAc/MeOH gradient and PTLC (20 × 20 cm, 0.25 mm) to give compounds **5** (1.3 mg; 5.2 × 10^−5^%) and **6** (0.9 mg; 3.6 × 10^−5^%).

#### 3.2.1. α-Angeloyloxy-6β-acetoxy-10β-hydroxy-9-oxo-furanoeremophilane (1)

Colorless crystal, mp 127–130 °C (hexane/EtOAc); ${\left[\alpha \right]}_{D}^{20}$ −28.2 (*c*, 0.82, CHCl_3_). IR (CHCl_3_) ν_máx_.: 3452, 1748, 1720, 1679, 1232 cm^−1^. EI-MS: 404 (1, M^+^), 345 (3), 260 (20), 262 (12), 178 (55), 136 (4), 91 (4), 83 (100), 57 (8), 55 (39). HR-EI-MS: 404.1838 (M^+^, C_22_H_28_O_7_; calculated for 404.1835). For ^1^H and ^13^C NMR data see Table 1.

#### 3.2.2. α-hydroxy-3α-angeloyloxy-10αH-9-oxo-furanoeremophilane (2)

White amorphous solid; ${\left[\alpha \right]}_{D}^{20}$ −37.5 (*c*, 0.59, CHCl_3_). IR (CHCl_3_) ν_máx_.: 3446, 1733, 1716, 1699, 1456 cm^−1^. EI-MS: 346 (3, M^+^), 246 (9), 228 (10), 213 (18), 191 (8), 163 (100), 135 (9), 105 (5), 91 (13), 83 (19), 77 (7), 55 (24). HR-EI-MS: 346.1785 (M^+^, C_20_H_26_O_5_; calculated for 346.1780). For ^1^H and ^13^C NMR data see Table 1 [12].

#### 3.2.3. β,10β-epoxy-6β-acetoxy-8α-hydroxy-eremophil-7(11)-en-8β,12-olide (3)

Colorless oil; ${\left[\alpha \right]}_{D}^{20}$ −72.8 (*c*, 0.25, CHCl_3_). IR (CHCl_3_) ν_máx_.: 3392, 1771, 1749, 1717 cm^−1^. EI-MS: 322 (0.4, M^+^), 298 (2), 280 (3), 262 (100), 244 (7), 216 (6), 142 (100), 124 (50), 95 (63). HR-EI-MS: 322.1425 (M^+^, C_17_H_22_O_6_; calculated for 322.1416). For ^1^H and ^13^C NMR data see Table 2.

#### 3.2.4. (+)-1(14)-en-maaliol (4)

Colorless oil; ${\left[\alpha \right]}_{D}^{20}$ +4.0 (*c*, 0.78, CHCl_3_). IR (CHCl_3_) ν_máx_.: 3421, 2930, 2868, 1653, 1457, 1375, 1152, 913, 889, 668 cm^−1^. EI-MS: 220 (5, M^+^), 205 (55), 187 (19), 177 (12), 162 (24), 159 (34), 147 (36), 133 (29), 121 (38), 119 (60), 105 (75), 93 (78), 91 (100), 79 (93), 69 (85). HR-EI-MS: 220.1831 (M^+^, C_15_H_24_O; calculated for 220.1827). For ^1^H and ^13^C NMR spectral data see Table 3.

#### 3.2.5. 9-O-angelylpetasinecine (hectorine) (5)

Colorless oil; ${\left[\alpha \right]}_{D}^{20}$ −62.86 (*c*, 0.07, CHCl_3_). EI-MS: 239 (9, M^+^), 222 (9), 190 (3), 188 (11), 140 (61), 122 (9), 111 (9), 83 (100), 70 (11), 68 (6), 55 (27). HR-EI-MS: 239.1512 (M^+^, C_13_H_21_NO_3_; calculated for 239.1521). ^1^H NMR (CDCl_3_, 500 MHz): δ_H_ 2.51 (1H, m, H_α_-C(1)), 4.23 (1H, t, *J* = 3.9 Hz, H_α_-C(2)), 3.42 (1H, dd, *J* = 4.0, 13.0 Hz, H_α_-C(3)), 3.03 (1H, d, *J* = 13.0, Hz, H_β_-C(3)), 3.50 (1H, t, *J* = 8.5 Hz, H_α_-C(5)), 2.98 (1H, m, H_β_-C(5)), 1.84 (1H, m, H_α_-(6)), 2.06 (1H, m, H_β_-C(6)), 1.80 (1H, m, H_α_-C(7)), 1.99 (1H, m, H_β_-C(7)), 3.90 (1H, m, H_α_-C(8)), 4.73 (1H, dd, *J* = 10.0, 11.5 Hz, H_d_-C(9)), 4.15 (1H, dd, *J* = 4.9, 11.5 Hz, H_u_-C(9)), 6.15 (1H, cc, *J* = 1.5, 7.2 Hz, H-(3’)), 1.99 (3H, d, *J* = 7.0 Hz, H-C(4’)), 1.90 (3H, quint., *J* = 1.6 Hz, H-C(5’)). ^13^C-NMR: δ(C) 46.3 (d, C-1), 73.2 (d, C-2), 61.6 (t, C-3), 56.9 (t, C-5), 27.2 (t, C-6), 27.9 (t, C-7), 66.7 (d, C-8), 60.3 (t, C-9), 169.2 (s, C-1’), 127.4 (s, C-2’), 139.8 (d, C-3’), 16.1 (q, C-4’), 20.7 (q, C-5’) [13].

#### 3.2.6. Rosmarinine (6)

As a white resin; ${\left[\alpha \right]}_{D}^{20}$ −51.1 (*c*, 0.09, CHCl_3_). EI-MS: 353 (5, M^+^), 282 (2), 227 (4), 180 (6), 156 (43), 154 (87), 138 (100), 122 (32), 98 (27), 82 (86), 81 (21), 55 (41). HR-EI-MS: 353.1835 (M^+^, C_18_H_27_NO_6_; calculated for 353.1838). ^1^H NMR (CDCl_3_, 500 MHz): δ(H) 2.54 (1H, m, H_α_-C(1)), 4.26 (1H, m, H_β_-C(2)), 3.15 (1H, dd, *J* = 7.3, 11.2 Hz, H_α_-C(3)), 2.94 (1H, dd, *J* = 7.9, 11.3 Hz, H_β_-C(3)), 3.33 (1H, t, *J* = 8.9 Hz, H_α_-C(5)), 2.60 (1H, m, H_β_-C(5)), 2.08 (1H, m, H_α_-C(6)), 2.27 (1H, m, H_β_-C(6)), 5.08 (1H, t, *J* = 2.9 Hz, H_α_-C(7)), 3.71 (1H, dd, *J* = 3.4, 7.8 Hz, H_α_-C(8)), 4.89 (1H, dd, *J* = 5.4, 12.6 Hz, H_d_-C(9)), 4.11 (1H, dd, *J* = 1.1, 12.6 Hz, H_u_-C(9)), 1.80 (1H, m, H_β_-C(13)), 2.27 (1H, m, H_a_-C(14)), 1.96 (1H, m, H_b_-C(14)), 1.34 (3H, s, H_α_-C(18)), 0.97 (3H, d, *J* = 6.7 Hz, H_α_-C(19)), 5.80 (1H, c, *J* = 7.1 Hz, H-(20)), 1.85 (3H, dd, *J* = 1.5, 7.2 Hz, H-C(21)). ^13^C-NMR: δ(C) 49.1 (d, C-1), 69.3 (d, C-2), 60.9 (t, C-3), 53.5 (t, C-5), 34.6 (t, C-6), 75.2 (d, C-7), 69.6 (d, C-8), 62.3 (t, C-9), 180.7 (s, C-11), 77.6 (s, C-12), 38.0 (d, C-13), 39.7 (t, C-14), 132.7 (s, C-15), 167.6 (s, C-16), 25.8 (q, C-18), 11.9 (q, C-19), 134.9 (d, C-20), 15.3 (q, C-21) [14].

#### 3.2.7. Crystal Structure Analysis

Intensity data, for both compounds, were collected at 293 K on an Oxford Diffraction Supernova dual Atlas CCD diffractometer, using Cu Kα (λ = 1.5418 Å) radiation. Data collection, cell refinement and data reduction were performed with the CrysAlisPRO [25] set of programs. The structure was solved by direct methods using SIR97 [26]. Refinements were performed with SHELXL-97 [27] using full-matrix least squares, with anisotropic displacement parameters for all the non-hydrogen atoms. The H-atoms were placed at calculated positions with C-H distances 0.95-1.00 Å and refined using a riding model. Calculations were mainly performed with WinGX [28] and molecular graphics were computed with PLATON [29].

X-ray crystal data: C_22_H_28_O_7_, Mw = 404.44, orthorhombic, space group, P2_1_2_1_2_1_, Z = 8, a = 8.7493(2), b = 13.4234, c = 37.0309(11) Å; V = 4349.1(2) Å^3^, μ(Cu Kα) = 0.76 mm^−1^, ρ_calc_ = 1.23 g.cm^−3^; S = 1.06, final R indices: R_1_ = 0.0678 and Rw = 0.1860 for 7134 observed from 8339 independent and 15715 measured reflections (θ_max_ = 70.99, I > 2σ(I) criterion and 536 parameters); maximum and minimum residues are and 0.30 and −0.24 e.Å^−3^ respectively. There are two independent molecules in the asymmetric unit with minor conformational differences between them. The absolute structure is based on the refinement of the Flack [30] (Flack 1983), x = 0.0 (3), parameter against 3610 CuKα Bijvoet pairs. The Hooft [31] analysis yielded y = 0.06(8) and P2 (true) = 1.000.

Crystallographic data (excluding structure factor tables) has been deposited with de Cambridge Crystallographic Data Center as supplementary publications no. CCDC1455588. Copies of the data can be obtained free of charge on application to The Director, CCDC, 12 Union Road, Cambridge CB1EZ, UK ((Fax: Int. + (1223) 336 033); e-mail: deposit@ccdc.cam.ac.uk)).

### 3.3. Insect Bioassays

*S. littoralis*, *M. persicae* and *R. padi* colonies were reared on an artificial diet [32], bell pepper (*Capsicum annuum*) and barley (*Hordeum vulgare*) plants, respectively. The plants are grown from seeds in pots with commercial substrate. The plants for rearing aphids are infected regularly (bell pepper plants with 4 leaves, barley plants of 10 cm length). The insect colonies and host plants were maintained at 22 ± 1 °C, > 70% relative humidity with a photoperiod of 16:8 h (L:D) in a growth chamber. 

Antifeedant bioassays: The upper surface of *C. anuum* and *H. vulgare* leaf disks or fragments (1.0 cm^2^) were treated with 10 μl of the test substance. The crude extracts and products were tested at an initial dose of 100 or 50 μg/cm^2^ respectively. Five Petri dishes (9 cm diam.) or twenty ventilated plastic boxes (2 × 2 cm) with two newly molted *S. littoralis* L6 larvae (≤24 h) or ten apterous aphid adults (24–48 h old) each were allowed to feed at room temperature for *S. littoralis* (<2 h) or in a growth chamber for the aphids (24 h, environmental conditions as above). Each experiment was repeated 2-3 times (SE < 10%) and terminated when the consumption of the control disks reached 65–75% for *S. littoralis* or after 24 h for aphids. The leaf disk area consumed was measured on their digitalized images (Image J, http://imagej.nih.gov/ij). Settling was measured by counting the number of aphids settled on each leaf fragment. Feeding or settling inhibition (%FI or %SI) was calculated as % FI/%SI = [1 − (T/C) × 100], where T and C are the consumption/settling of treated and control leaf disks, respectively. The antifeedant effects (% FI/SI) were analyzed for significance by the nonparametric Wilcoxon signed-rank test. Extracts and compounds with an FI/SI ≤ 75% were further tested in a dose-response experiment (3–4 serial dilutions) to calculate their relative potency (EC_50_, the effective dose to give a 50% feeding/settling reduction) from a linear regression analysis (% FI/SI on Log-dose) [33].

## 4. Conclusions

*Senecio fistulosus* is characterized by their content in sesquiterpenes (furanoeremophilanes, eremophilanolides and maaliane type) and pyrrolizidine alkaloids. The antifeedant properties of ethanolic, non-alkaloidal, alkaloidal extracts and compounds have been studied. Most of the insect antifeedant effects were found in the ethanolic and non-alkaloidal extracts, containing mainly sesquiterpenes with low amounts of PAs. The isolated furanoeremophilanes sesquiterpenes type had structure-dependent antifeedant effects. In addition to their antifeedant action, these sesquiterpenes could play a role in insect-plant interactions.

## Figures and Tables

**Table 1:** ^1^H (500 MHz) and ^13^C (125 MHz) NMR data of compounds 1 and 2 in CDCl_3_.

Position	1		2	
	δ_H_ in ppm, Multiplicity, *J* (in Hz)	δ_C_ in ppm	δ_H_ in ppm, Multiplicity, *J* (in Hz)	δ_C_ in ppm
1β	4.84 *br* s	74.5 d	4.25 ddd (4.8, 9.7, 11.7)	65.5 d
2α	2.30 m	20.7 t	1.50 q (11.8)	39.1 t
2β	1.64 m		2.47 m	
3α	1.40 m	23.9 t	-	71.4 d
3β	2.32 m		4.90 dt (4.6, 11.5)	
4α	1.65 m	32.3 d	1.84 m	46.9 d
5	-	50.2 s	-	43.9 s
6α	7.03 s	68.6 d	2.52 d (16.6)	35.9 t
6β	-		2.71 d (16.6)	
7	-	139.4 s	-	138.1 s
8	-	145.9 s	-	146.5 s
9	-	186.9 s	-	188.7 s
10β	-	79.8 s	2.42 d (9.8)	60.6 d
11	-	121.8 s	-	121.7 s
12	7.41 *br* s	146.9 d	7.41 s	145.8 d
13	1.91 d (1.1)	8.3 q	1.99 d (1.0)	7.8 q
14	0.98 s	15.5 q	0.88 s	14.3 q
15	1.17 d (7.5)	16.0 q	0.98 d (6.7)	10.5 q
1’	-	165.7 s	-	167.6 s
2’	-	126.6 s	-	128.1 s
3’	5.92 qq (1.5, 7.2)	140.5 d	6.05 qq (1.4, 7.2)	137.8 d
4’	1.88 dq (1.6, 7.4)	15.8 q	1.97 dq (1.5, 7.2)	15.8 q
5’	1.55 quint. (1.5)	19.9 q	1.88 quint. (1.5)	20.6 q
OCOCH_3_	2.19 s	20.9 q	-	-
OCOCH_3_	-	170.9 s	-	-
OH-10	3.98 *br* s	-	-	-

**Table 2:** ^1^H (500 MHz) and ^13^C (125 MHz) NMR data of compounds **3** and **7** * in CDCl_3_.

Position	3		7 *	
	δ_H_ in ppm, Multiplicity, *J* (in Hz)	δ_C_ in ppm	δ_H_ in ppm, Multiplicity, *J* (in Hz)	δ_C_ in ppm
1β	3.18 d (4.6)	62.7 d	3.14 d (4.3)	63.0 d
2α	1.96 dd (6.8, 10.8)	20.3 t	2.04 m	21.0 t
2β	2.04 dd (5.9, 10.5)		2.21 m	
3α	1.36 dc (3.5, 9.4)	23.9 t	1.38 m	24.2 t
3β	1.63 m		1.61 m	
4α	1.62 m	32.5 d	1.62 m	33.0 d
5	-	43.4 s	-	43.5 s
6α	5.92 c (1.8)	73.8 d	5.69 t (1.7)	74.3 d
7	-	155.0 s	-	153.9 s
8	-	101.3 s	-	104.4 s
9α	1.79 d (13.6)	43.4 t	1.80 d (13.6)	43.6 t
9β	2.31 d (13.6)		2.27 d (13.6)	
10	-	60.9 s	-	61.0 s
11	-	124.6 s	-	126.8 s
12	-	170.8 s	-	170.9 s
13	1.87 d (1.8)	8.2 q	1.92 d (1.2)	8.6 q
14	1.09 s	14.5 q	1.09 s	14.5 q
15	1.04 d (7.2)	16.1 q	1.03 d (7.0)	16.5 q
OMe-8	-	-	3.23 s	50.9 q
OCOCH_3_	2.20 s	20.9 q	2.21 s	21.0 q
OCOCH_3_	-	170.6 s	-	170.3 s

* Source: Reina et al. [2].

**Table 3:** ^1^H (500 MHz), ^13^C (125 MHz) and HMBC NMR data of compound **4** in CDCl_3_.

Position	δ_H_ in ppm, Multiplicity, *J* (in Hz)	δ_C_ in ppm	HMBC
1	-	153.6 s	-
2a	2.42 ddd (1.3, 6.3, 13.3)	39.1 t	C-1, C-4, C-10, C-14
2b	2.05 m		
3a	1.77 m	41.9 t	C-2, C-4
3b	1.56 d		
4	-	81.1 s	-
5α	1.32 m	54.6 d	C-1, C-4, C-6, C-10, C-11
6β	0.47 dd (9.6, 11.4)	30.1 d	C-4, C-8, C-11, C-13
7β	0.71 ddd (6.1, 9.5, 11.4)	27.7 d	C-5, C-11, C-13
8a	1.98 m	24.9 t	-
8b	1.01 m		
9a	1.90 m	26.9 t	C-5, C-10
9b	1.63 m		
10β	2.20 m	53.6 d	C-1, C-2, C-5, C-6, C-14
11	-	20.4 s	-
12	1.06 s	28.8 q	C-7, C-6, C-11, C-13
13	1.04 s	16.5 q	C-7, C-6, C-11, C-12
14a	4.69 t (1.6)	106.4 t	C-2, C-10
14b	4.63 q (1.7)		
15	1.28 s	26.2 q	C-3, C-4, C-5

**Table 4:** Antifeedant activity of *S. fistulosus* compounds **1**, **2**, **4**.

Compound	*S. littoralis*	EC_50_ (μg/cm^2^) ^b^	*M. persicae*	EC_50_ (μg/cm^2^) ^b^
	%FI (50 μg/cm^2^) ^a^		%SI ^b^ (50 μg/cm^2^) ^a^	
**1**	65 ± 6 *		52 ± 7	
**2**	83 ± 6 *	0.64 (0.36-1.16)	52 ± 7	
**4**	64 ± 7 *		90 ± 3 *	0.97 (0.71–1.32)

^a^ %FI/%SI = [1 − (T/C)] × 100, where T and C are the consumption/settling of treated and control leaf disks, respectively. ^b^ Effective antifeedant dose (EC_50_) and 95% confidence (lower, upper). * *p* < 0.05, Wilcoxon paired test.

**Table 5:** Antifeedant structure-activity relationships of *Senecio* furanoeremophilanes against *S. littoralis* and *M. persicae*.

Compound	Substituent				*S. littoralis*	*M. persicae*
	C-1	C-3	C-6	C-10	%FI ^c^ (EC_50_) ^d^	%SI ^c^ (EC_50_) ^d^
**1**	α-OAng	H_2_	β-OAc	β-OH	65.0	52.0
**2**	α-OH	α-OAng	H_2_	α-H	83.0 (0.64)	52.0
**8 ^a^**	Δ^1^	H_2_	β-OAng	Δ^10^	32.0	67.0
**9 ^a^**	Δ^1^	H_2_	β-OH	Δ^10^	62.0	71.0
**10 ^a^**	Δ^1^	H_2_	β-OCOCH_2_CH_3_	Δ^10^	45.0	75.0
**11 ^b^**	H_2_	H_2_	β-OAc	α-H	51.0	74.0 (21.9)
**12 ^b^**	H_2_	H_2_	β-OTigl	α-H	65.0	74.0 (12.2)

^a^ Compounds **8–10** from Domínguez et al. [11]. ^b^ Compounds **11, 12** from Reina et al. [2]. ^c^ %FI / %SI values at 50μg/cm^2^. ^d^ Effective antifeedant dose (μg/cm^2^).
